# Difference analysis of intestinal microbiota in patients in the intensive care unit using different sampling methods: a systematic review and meta-analysis

**DOI:** 10.3389/fmicb.2025.1723862

**Published:** 2026-01-23

**Authors:** Songlin Qiu, Binyang Zheng, Juan Pan, Sufei Yu, Jiao Qian, Tao-Hsin Tung, Bo Shen

**Affiliations:** 1 Department of Clinical Laboratory, Taizhou Hospital of Zhejiang Province Affiliated to Wenzhou Medical University, Linhai, China; 2 Key Laboratory of System Medicine and Precision Diagnosis and Treatment of Taizhou, Taizhou, China; 3 Evidence-based Medicine Center, Taizhou Hospital of Zhejiang Province, Wenzhou Medical University, Linhai, China

**Keywords:** diversity, dysbiosis, intensive care center, intensive care unit, microbiome

## Abstract

**Background:**

The normal intestinal microbiota undergoes rapid and notable changes in patients in the intensive care unit (ICU) because of factors such as host physiological stress, changes in gastrointestinal function, and antibiotic exposure. Different specimen types are used for intestinal microbial analysis because of sampling difficulties. Therefore, this study conducted a meta-analysis to investigate changes in the intestinal microbiota of patients admitted to the ICU and whether using different specimen types affects microbiota analysis.

**Methods:**

A systematic review was conducted encompassing studies published in electronic databases up to May 1, 2024. We included 11 studies that compared the abundance and diversity of the gut microbiota between ICU patients and healthy cohorts (HC). A standardized mean difference (SMD) meta-analysis using random effects models was performed to quantify microbial differences, including an assessment of various sampling methods.

**Results:**

After ICU admission, the intestinal microbiota of patients differed significantly from that of the normal population, showing lower diversity and richness. A significant difference in beta diversity was also observed. Specifically, the relative abundances of Proteobacteria and Fusobacteria were elevated in ICU patients, while Firmicutes abundance was diminished. Crucially, the comparison of stool versus rectal swab specimens demonstrated no significant difference in the measured alpha diversity of the gut microbiota.

**Conclusion:**

The early intestinal microbiota of patients in the ICU differed from that of healthy individuals. A comprehensive understanding of the early changes in the intestinal microbiota of patients in the ICU can help formulate prevention and treatment strategies. Furthermore, using feces and swab samples for analysis did not significantly affect the diversity of the intestinal microecology. Therefore, rectal swabs may be an attractive method for sampling the gut microbiota and metabolome.

**Systematic review registration:**

PROSPERO Registration number is CRD42022385146 (Available from: https://www.crd.york.ac.uk/PROSPERO/view/CRD42022385146).

## 1 Introduction

Gut microbes play an essential role in human disease process and progression (Ding et al., 2019). Advances in research tools and techniques have yielded new insights into microbe–host interactions (Martinez et al., 2017; Afzaal et al., 2022). In clinical settings, antimicrobial therapy remains crucial for managing infectious diseases, particularly among critically ill patients (Arulkumaran et al., 2020). Yet, the rise of multidrug-resistant bacteria complicates effective antibiotic and anti-infection treatments in severely ill individuals (Kollef et al., 2017).

Patients in ICUs often experience substantial changes in the complexity of their gut microbiota. Patients develop their unique gut microbiome within 48 h of intensive care unit (ICU) admission. Critical illness and intensive care directly drive alterations in gut microbiome composition. Compared with healthy individuals, ICU patients typically exhibit diminished microbial diversity and a shift toward pathogenic dominance; the abundance of *Enterococccus* and other pathogens is substantially elevated (Cho et al., 2024). These dysbiotic shifts are particularly prevalent in patients suffering from sepsis (Agudelo-Ochoa et al., 2020; Howard et al., 2017). Gut dysbiosis is recognized as an independent risk factor for increased 28-day mortality in critically ill patients, whereas increased alpha diversity(*α*-diversity) is associated with reduced in-hospital mortality (Garcia et al., 2022; McDonald et al., 2016; Salameh et al., 2023; Sansom et al., 2023). α-diversity, which reflects the richness and evenness of microbial communities, is crucial for comparing microbial composition across different patient groups.

Fecal specimens are the reference standards for gut microecology studies (Ravi et al., 2019). However, because obtaining stool samples from patients in the ICU is often challenging owing to complications such as constipation and intestinal obstruction, rectal swabs have become the most common method of sampling the intestinal microbiota in these patients (Fair et al., 2019; Budding et al., 2014; Bassis et al., 2017). However, the two different sampling methods of feces and swabs do not differ substantially in subsequent intestinal microbiota analysis (Bansal et al., 2018). Specimens obtained using the two sampling methods were similar in the richness and evenness of the gut microbiome, and no difference in *α*-diversity was observed (Short et al., 2021; Schlebusch et al., 2022; Bokulich et al., 2019). However, some differences between stool and swab specimens have been reported, with stool specimens having a lower diversity of gut microbiota than swab specimens (Turner et al., 2022; Kwon et al., 2021).

In the late stages of ICU admission, gut microbiota dysbiosis can be attributed to parenteral feeding, antibiotic use, and mechanical ventilation. However, the rapid and dynamic changes in gut microbiota in the early stages after ICU admission remain unclear. Second, when characterizing the gut microbiota of patients in the ICU, clear reproducibility of the differences in gut microbiota is lacking between swabs and stools across different studies.

Therefore, this systematic review and meta-analysis evaluated early gut microbiota alterations in ICU patients by assessing differences in microbial diversity and relative abundance compared with healthy individuals, based on data from both stool and rectal swab samples. These findings may contribute to identifying microbiota-related pathways that could be targeted in future ICU interventions.

## 2 Materials and methods

### 2.1 Search strategy

**Figure 1:**
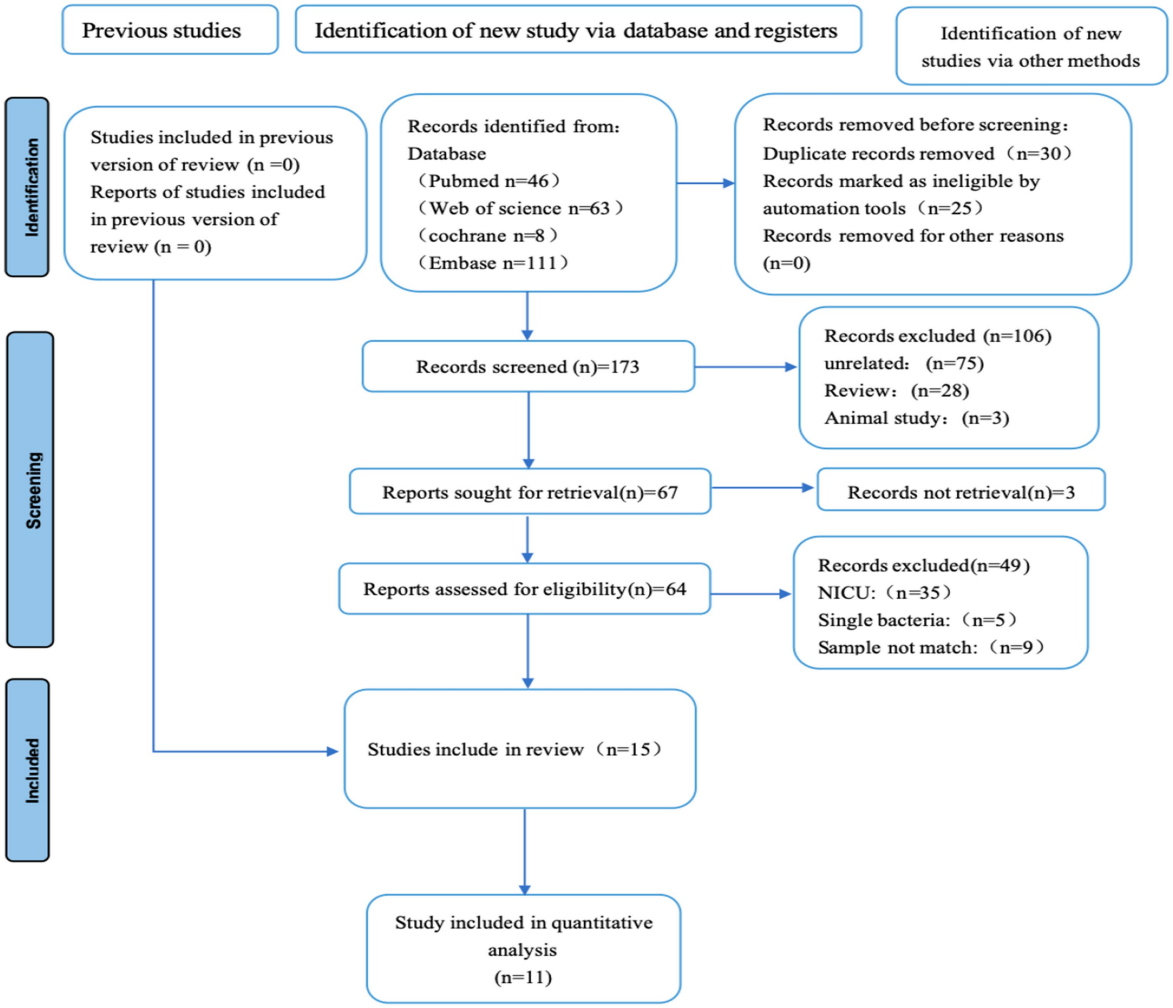
PRISMA study flow chart.

This systematic review was conducted according to the Preferred Reporting Items for Systematic Reviews and Meta-Analyses guidelines (Page et al., 2021). PubMed, Web of Science, Cochrane, China National Knowledge Internet, and Embase were searched for cases for cross-sectional analysis (Figure 1). All literature from database inception to May 1, 2024, was screened. The searches utilized both MeSH terms and free-text keywords. No language restrictions were applied to the literature retrieval. There were no language restrictions for literature retrieval, and the search process is shown in (Supplementary Table 1). This systematic review is registered with PROSPERO (CRD42022385146).

### 2.2 Inclusion and exclusion criteria

Studies were included if they met the criteria for study type, participant characteristics, applied intervention, and outcome measures. 16S analysis of samples was preferred to identify species without the need for cultivation. Only studies published since 2010 were included owing to the recent availability of 16S sequencing technology. The cohort included patients with an ICU admission time longer than 24 h, while excluding those in the Neonatal Intensive Care Unit (NICU) and the Emergency Intensive Care Unit (EICU). This approach was implemented to minimize biological heterogeneity, as the physiological and microbial colonization processes in the excluded groups fundamentally differ from those in adult ICU patients (Christoff et al., 2020). A minimum Intensive Care Unit (ICU) stay of 24 h was selected to exclude patients admitted for brief observation and to ensure that the included patients received sufficient exposure to critical care interventions. Longitudinal data from existing studies support that significant alterations in gut microbiota occur within 24 h to several days after ICU admission (Ojima et al., 2022; Wozniak et al., 2024). The included studies used various methods for sample collection and processing. Two studies excluded patients with concomitant perianal diseases. Rectal swabs were collected by inserting a cotton swab 1 to 2 centimeters from the anal verge and gently wiping the rectal mucosal surface. Stool specimens were usually collected in sterile polypropylene containers. After collection, the samples were stored frozen in a freezer until DNA extraction, when they were thawed for processing.

### 2.3 Data extraction and quality assessment

Available data were extracted from all selected trials; Drs. Qiu and Zheng extracted all the data while Dr. pan checked the accuracy of the data entry. Differences were discussed, and a consensus was reached. We extracted aggregate data from the included studies, such as the mean patient age, median length of ICU stay, and the percentage of patients receiving different sequencing methods. We attempted to contact the original authors to request missing data. The extracted data were quantitative and were summarized, with a meta-analysis performed on applicable numerical data. Two authors independently used the Newcastle–Ottawa Scale to assess the quality of the included studies (Stang, 2010). The selection bias is assessed using funnel plots (Supplementary Figure 1).

### 2.4 Data processing and statistical analysis

We extracted intergroup comparisons of relative gut microbial abundance and alpha and beta diversity(*β*-diversity)indices for quantitative and qualitative summary. *α*-diversity was calculated to assess the richness and evenness of the gut microbiota. Specifically, Chao1 index was used to estimate observed richness, Shannon index to quantify diversity, and Simpson index to evaluate evenness. These indices were treated as primary outcomes in our meta-analysis. The mean (M) and standard deviation (SD) of *α*-diversity indices and relative microbial abundance were extracted from the included studies. If only the median and interquartile range were reported, they were converted to M and SD using a web-based tool.^1^ If necessary, numerical data were extracted from the image using a WebPlot digitizer (v.4.4). The SMD and 95% confidence interval (CI) of the above indicators in the patients in the ICU and HCs were calculated. Heterogeneity among studies was assessed using Cochrane’s *Q* test (with significance set at *p* < 0.10) and quantified by the *I*^2^ statistic. A fixed-effects model was applied if *I*^2^ ≤ 50%, otherwise, a random-effects model was used to pool effect sizes. For full reproducibility, the complete dataset (including all extracted mean, standard deviation (s), and sample size (n) values) is available as Supplementary Table 2. The R script utilized for all meta-analysis calculations and figure generation is available in Supplementary material (R script file).

### 2.5 Risk of bias

The following data were collected: study characteristics (country, setting, study design, and period), study population (age range, race/ethnicity, and inclusion criteria), exposure information (i.e., factors that may have influenced the composition of the documented gut microbiome), length of ICU stay, sampling time, analytical methods, and diversity measures.

## 3 Results

### 3.1 Search results

A total of 228 records were identified through the initial search. Of these, 213 were excluded based on the pre-defined criteria (e.g., duplicates, studies conducted in neonatal ICUs, single-strain investigations, or those not meeting the required sampling criteria). Studies without a control group were further excluded, leaving 11 studies eligible for inclusion in the final meta-analysis.

### 3.2 Characteristics of included studies

The 11 included studies were conducted in Australia, China, Italy, Finland, Japan, and Canada. The cohort criteria of patients in the ICU were patients older than 18 years, an ICU stay of 2–30 days, antibiotics were not used or were used in small amounts upon admission and were only used after entering the ICU ward. Studies were excluded as follows: patients younger than 18 years, diseases associated with perianal infection, invalid specimens, and stool samples, excluding those obtained by enema. All samples were analyzed using 16S RNA sequencing or whole-genome sequencing, and the samples were stored at −30 °C, −78 °C or −80 °C (Table 1).

**Table 1:** The characteristics and quality of included study.

Study	Region-state	Object	Case (Sample/number)	Control (Sample /number)	Inclusion criteria of ICU	Analysis methods	Store condition	Score	Convenience sampling
Chernevskaya et al. (2020)	Australia	ICU vs. HC	Feces /47	Feces /23	Days after admission to ICU > 3	16S RNA Sequence	−30 °C	7/9	Yes
Zhang et al. (2019)	China	ICU vs. HC	Feces /16	Feces /10	Days after admission to ICU > 2	16S RNA Sequence	−80 °C	8/9	Yes
Gaibani et al. (2021)	Italy	ICU vs. HC	Feces /69	Feces /69	Consecutive adult (≥18 years)	16S RNA Sequence	−80 °C	6/9	Yes
Wan et al. (2018)	China	ICU vs. HC	Feces /15	Feces /15	Consecutive adult (≥18 years)	16S RNA Sequence	−80 °C	7/9	Yes
Ojima et al. (2022)	Japan	ICU vs. HC	Swabs /71	Swabs /9	Days after admission to ICU > 3	16S RNA Sequence	−78 °C	7/9	Yes
Mazzarelli et al. (2021)	Japan	ICU vs. HC	Swabs /6	Swabs /8	Consecutive adult (≥18 years)	16S RNA Sequence	−80 °C	7/9	Yes
Zaborin et al. (2014)	USA	ICU vs. HC	Feces /14	Feces /5	Days after admission to ICU > 3	16S RNA Sequence	−80 °C	7/9	Yes
Long et al. (2023)	China	ICU vs. HC	Feces /16	Feces /10	Days after admission to ICU > 2	ICU vs. HC	−80 °C	8/9	Yes
Schlebusch et al. (2022)	Australia	ICU vs. ICU	Feces /18	Swabs /18	Consecutive adult (≥18 years)	WGSM	−80 °C	8/9	Yes
Fair et al. (2019)	USA	ICU vs. ICU	Feces /39	Swabs /132	Days after admission to ICU > 3	16S RNA Sequence	−80 °C	8/9	Yes
Bansal et al. (2018)	Canada	ICU vs. ICU	Feces /15	Swabs /55	Consecutive adult (≥18 years)	16S RNA Sequence	−80 °C	8/9	Yes

Study quality was assessed using the Newcastle-Ottawa Scale (NOS) (Wells et al., 2025), which consists of three domains: Selection (0–4 points), Comparability (0–2 points), and Outcome (0–3 points).studies scoring 7–9 points were considered low risk of bias, 4–6 points moderate risk, and ≤3 points high risk (Wells et al., 2025). HC, healthy controls; ICU, intensive care unit; WGSM, whole genome sequencing.

### 3.3 Characteristics of the included population

The enrolled ICU patients were all critically ill, with some cohorts specifically reporting severe pneumonia (Chernevskaya et al., 2020; Zhang et al., 2019). Acute critically ill patients consistently exhibited significant intestinal microbiota dysbiosis compared to healthy controls (HCs), primarily characterized by reduced abundance and diversity, with the microbiota community structure being significantly altered (*p* = 0.05). Gaibani et al. (2021) and Mazzarelli et al. (2021) included patients admitted to the ICU due to respiratory failure caused by COVID-19. Compared to HCs, a significant decrease in *α*-diversity was observed in patients with COVID-19 (*p* = 0.08), and the Chao1 index was reduced. Wan et al. (2018) found that septic shock patients exhibited significantly reduced bacterial diversity (*p* < 0.05) compared to HCs, with a higher abundance of Proteobacteria and Fusobacteria. Ojima et al. (2022) included 71 patients with mechanical ventilation, and broad-spectrum antibiotics caused intestinal microbiota diversity loss in the acute phase of ICU hospitalization. Zaborin et al. (2014) included ICU patients with various underlying conditions. Among those with long-term ICU stays, over 80% showed reduced stool microbial diversity (Chao1 < 50), and approximately 50% exhibited extremely low diversity (Chao1 < 10). Long et al. (2023) included non-septic patients in an ICU population. They found that the Chao1 index and Shannon diversity were significantly lower than in healthy controls, and significant differences were observed between Firmicutes and Proteobacteria. These eight studies all compared the diversity and abundance of the microbiota between patients admitted to the ICU and a normal healthy population for different reasons.

Rectal swabs can be used as a supplement to other sample types to analyze the intestinal microbiome in critical illnesses. Bansal et al. (2018) compared rectal swabs and fecal samples in ICU patients and found no significant differences in microbial community composition (PERMANOVA based on Bray–Curtis, *p* = 0.69; UniFrac, *p* = 0.86). McDonald et al. (2016) evaluated mechanically ventilated ICU patients and reported significant differences in *α*- and *β*-diversity between stool and rectal swab samples at both the phylum and genus levels after adjusting for potential confounders. Arulkumaran et al. (2020) compared swabs and feces in critically ill patients and observed no significant differences in gene- or category-level detection (*p* = 0.36 and *p* = 0.50, respectively).

### 3.4 Alpha diversity

*α*-diversity was used to assess the richness and evenness of the gut microbiota. Differences in α-diversity between ICU patients and healthy controls were analyzed using Chao1, ACE (Abundance-based Coverage Estimator), Shannon, Simpson, and PD (Phylogenetic Diversity)-whole-tree indices. Among the 11 included studies, eight used stool samples or swab specimens to analyze the differences in intestinal microbiota between the same group (patients in the ICU) and HCs. The other three studies analyzed the same group (patients in the ICU) using two different specimens (swabs and feces) to analyze the *α*- diversity of the intestinal microbiota.

**Figure 2:**
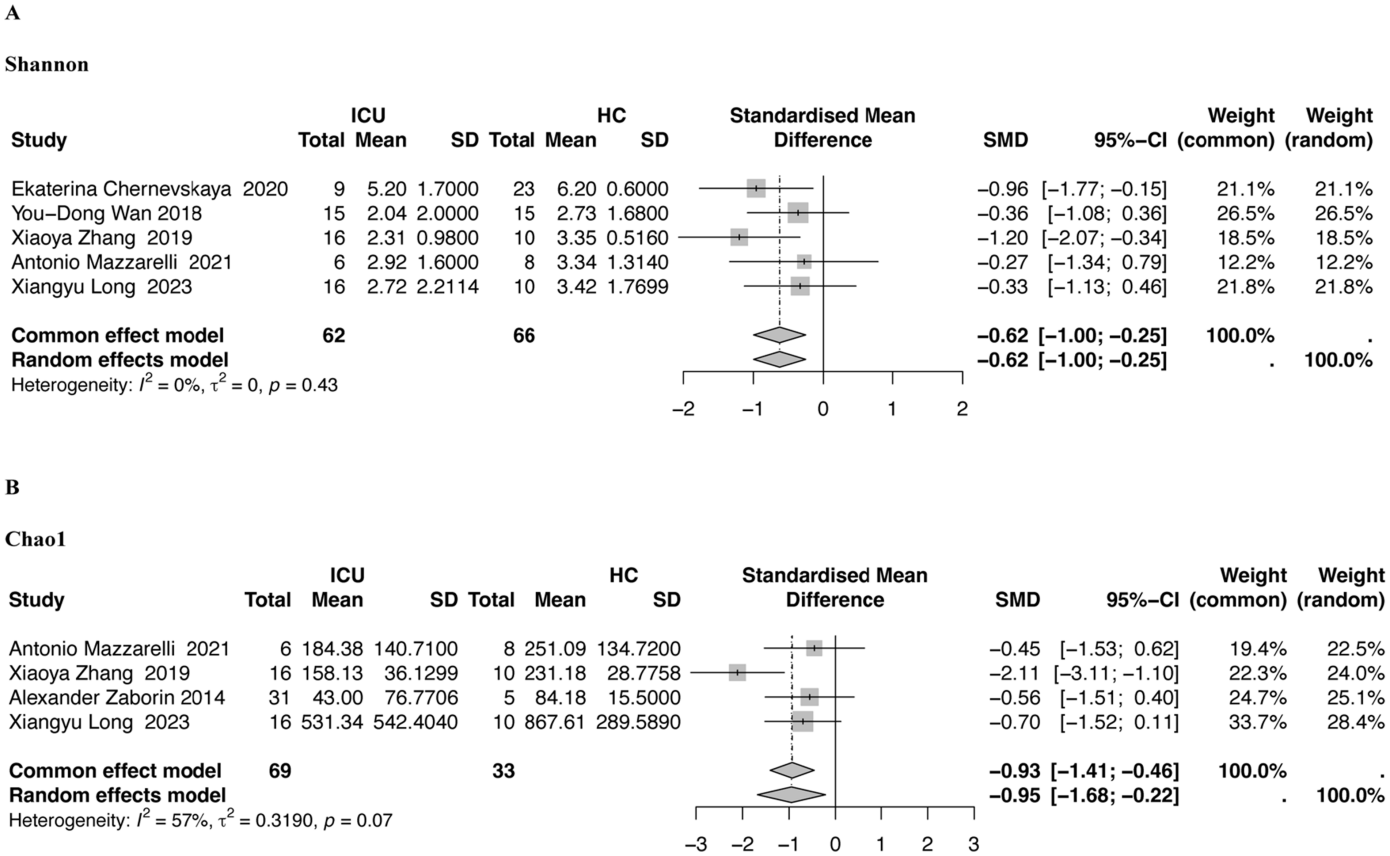
Forest plots of gut microbiota *α*-diversity in the stool samples of patients in the ICU compared with that of HCs. **(A)** Shannon index, **(B)** Chao1 index. ICU, intensive care unit; HC, healthy controls, *I^2^,* I-squared statistic used to quantify heterogeneity among studies.

Regarding community diversity, the Shannon index was provided for the five studies in Figure 2A (SMD = −0.62, 95% CI, −1.00 to −0.25, *p* = 0.43, *I^2^* = 0%). The diversity of the intestinal community in the stool samples of patients in the ICU decreased, and the difference was apparent when compared to the HC group; however, the difference was not statistically significant.

Regarding community richness, four studies provided Chao1 (SMD = −0.95, 95% CI, −1.68 to −0.22, *p* = 0.07, *I^2^* = 57%), and the intestinal microbiota species of specimens from swabs from patients in the ICU, which differed significantly from that of the HC group (Figure 2B).

**Figure 3:**
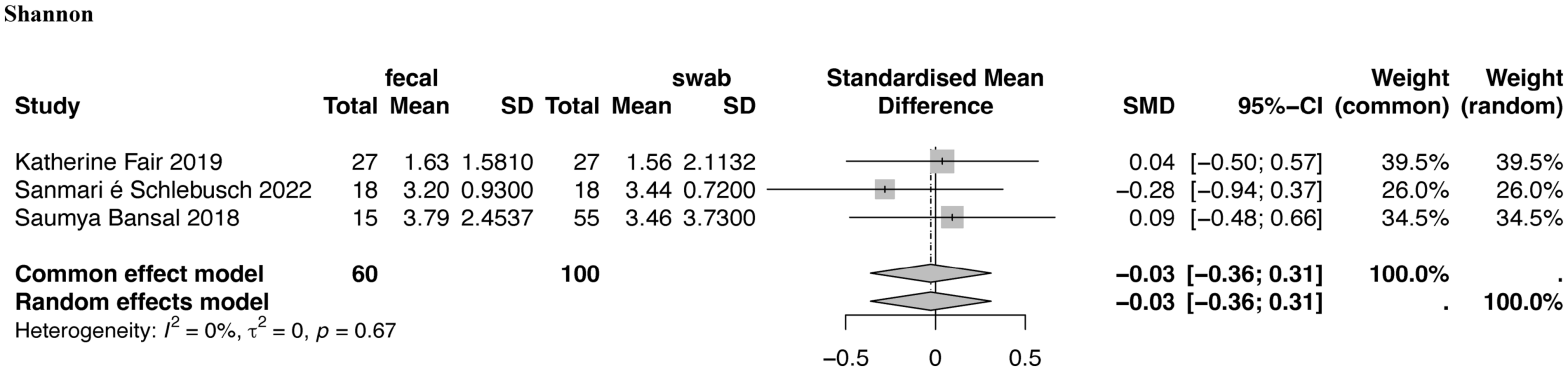
Forest plot of gut microbiota α-diversity in stool and swab samples from patients in the ICU. Shannon index.

Regarding community diversity, the Shannon index was provided in three studies (SMD = −0.12, 95% CI, −0.39–0.16, *p* = 0.67, *I^2^* = 0%). No significant difference was observed in the community between fecal and swab-derived specimens from patients in the ICU (Figure 3).

Regarding community diversity, three studies directly comparing fecal and rectal swab samples in ICU patients reported Shannon index values (Fair et al., 2019; Bansal et al., 2018; Schlebusch et al., 2022). The pooled results showed no significant difference between the two sampling methods [SMD = −0.03; 95% CI, (−0.36, 0.31); *p* = 0.67; *I^2^* = 0%], indicating that fecal and swab samples provide comparable estimates of *α*-diversity in ICU patients (Figure 3). These studies did not include healthy controls, as they focused on comparing sample types to assess α-diversity between fecal and swab samples in ICU patients. No significant differences were observed.

### 3.5 Beta diversity

Five studies reported *β*-diversity in patients in the ICU and HCs (Table 2). Four studies (Ojima et al., 2022; Zhang et al., 2019; Gaibani et al., 2021; Mazzarelli et al., 2021) used principal coordinate analysis, and one study (Wan et al., 2018) used non-metric multidimensional scaling. The β-diversity of the ICU group and the HC differed significantly (Table 2).

**Table 2:** α-Diversity and *β*-diversity between ICU and HC.

Study	α-diversity	β-diversity
Chernevskaya et al. (2020)	Shannon	/
Zhang et al. (2019)	Shannon/ACE/Chao1/Simpson	PCoA
Gaibani et al. (2021)	Simpson	PCoA
Wan et al. (2018)	Shannon	NMDS
Ojima et al. (2022)	/	PCoA
Mazzarelli et al. (2021)	Chao1/Shannon	PCoA
Zaborin et al. (2014)	Chao1	/
Long et al. (2023)	Chao1/Shannon/OTU number	/

PCoA, Principal Coordinates Analysis; NMDS, Non-Metric Multidimensional Scaling.

### 3.6 Diversity of phylum-level taxa

Seven studies were included to compare the differences in intestinal microbiota between patients in the ICU and HCs, and five studies specifically described the main differences at the phylum level. The abundance of Proteobacteria (Wan et al., 2018; Mazzarelli et al., 2021) and Fusobacteria (Zhang et al., 2019; Wan et al., 2018) was higher in patients in the ICU than in HCs, whereas the abundance of Firmicutes (Zhang et al., 2019; Zaborin et al., 2014) gradually decreased with time in the ICU and was replaced by Proteobacteria. The ratio of Bacteroidetes to Firmicutes was unbalanced within 7 days after admission, and the mortality rate was higher when the ratio was > 8 or < 1/8 (Ojima et al., 2022).

## 4 Discussion

A limited number of studies were included in the present meta-analysis. The funnel plot (Supplementary Figure 1) was used for a preliminary, exploratory assessment of symmetry and should not be interpreted as a formal evaluation of publication bias. This limited number of studies also restricts in-depth assessment of differences between stool and swab samples in ICU patients, as well as the evaluation of alpha and *α*- diversity.

Furthermore, In the present study, no significant differences in key alpha and α- diversity measures were observed between swab and stool samples in HCs. Collecting stool samples regularly during the ICU stay is not feasible, and rectal swabs can help solve this limitation. Research indicates that for individuals with colorectal polyps, swab samples provide a distinct and potentially richer view of the local microbiome compared to traditional stool samples. The swab method appears to capture different microbial characteristics and shows a higher relative abundance of microbes than what is measured in the stool (Jones et al., 2018). Two studies (Fair et al., 2019; Schlebusch et al., 2022) used stool and swab samples collected before and after the study to detect differences in intestinal microbiota diversity in the ICU population. Systematic differences were still observed in *α*- diversity after adjusting for potential confounding factors in the ICU population (such as time) in one study (Fair et al., 2019). Simultaneously, the other study didnot observe significant differences in the overall diversity of the intestinal microbiota (Schlebusch et al., 2022). However, the present meta-analysis was biased because of the limited number of included studies. For example, the small number of samples included in the study and the fact that the patients were specific cohorts (Fair et al., 2019; Bansal et al., 2018; Schlebusch et al., 2022) made it challenging to assess specific real differences.

Our meta-analysis results demonstrate that the gut microbiota of ICU patients exhibits the classic pattern of dysbiosis at the phylum level, with a significant reduction in commensal phyla such as Firmicutes and a concomitant increase in potential pathogens such as Proteobacteria and Fusobacteria (Zhang et al., 2019; Wan et al., 2018; Mazzarelli et al., 2021; Zaborin et al., 2014). This microbial structure shifts toward a pro-inflammatory and aerobic dominance, which is a hallmark of host stress, antibiotic exposure, and nutritional disruption in the ICU setting (Oami et al., 2019; Chanderraj et al., 2023). The structural imbalance carries critical prognostic significance: pivotal studies have demonstrated that the extreme imbalance of the Bacteroidetes/Firmicutes (B/F) ratio within 7 days of ICU admission is significantly associated with mortality, with patient mortality significantly increasing when the B/F ratio is > 8 or < 1/8 (Ojima et al., 2022; Ojima et al., 2016). The significant reduction in commensal Firmicutes revealed by our meta-analysis is consistent with the B/F ratio skewing phenomenon proposed by previous scholars, and powerfully reflects the microbiological signature of this mortality-associated B/F imbalance. This finding reflects the typical characteristic of pathogen dominance and commensal depletion in the ICU gut, further demonstrating that specific microbial abundance changes are closely related to mortality and poor clinical outcomes in ICU patients (Evans et al., 2023). These studies emphasize that monitoring the dynamic changes in the gut microbiota of ICU patients, combined with clinical intervention measures, may help improve prognosis. Therefore, the early identification and intervention of gut dysbiosis may represent an important direction for future ICU therapeutic strategies.

This study also had many confounding factors. First, the small sample size restricted in-depth analyses of genus- and species-level differences among ICU patients and prevented assessment of the dynamic relationship between disease severity and intestinal microbiota (Chernevskaya et al., 2020; Gaibani et al., 2021; Mazzarelli et al., 2021). Second, widespread antibiotic use and variations in antibiotic types and regimens, along with differing specimen collection time points, limited the ability to evaluate longitudinal microbiota changes consistently (Wan et al., 2018). Second, widespread antibiotic use and variations in antibiotic types and regimens, along with differing specimen collection time points, limited the ability to evaluate longitudinal microbiota changes consistently (Mazzarelli et al., 2021). Fourth, nonstandardized diagnosis and treatment protocols for bacterial superinfection further constrained follow-up (Gaibani et al., 2021). Fourth, nonstandardized diagnosis and treatment protocols for bacterial superinfection further constrained follow-up (Fair et al., 2019). Therefore, analyses of intestinal microbiota must account for sample type, timing of collection, and patient-specific clinical factors.

We acknowledge several limitations inherent to this systematic review and meta-analysis. A significant portion of these limitations stem directly from the inconsistent and incomplete reporting of key clinical and methodological variables across the included primary studies. For example, we were unable to adjust for potential confounding covariates, such as age, sex, comorbidities, or antibiotic exposure, due to this incomplete reporting. Consequently, the observed differences in gut microbiota composition between ICU patients and healthy controls may be influenced by these unmeasured factors. Furthermore, the small dataset available for sampling methods, the lack of integration of available functional or metabolomic findings, and the prevalence of cross-sectional or single-timepoint studies in the literature collectively pose significant restrictions on the depth and generalizability of our meta-analysis.

To enhance the robustness and reliability of future research, the standardization of methodology and reporting of gut microbiota studies becomes critically important. Given the current methodological heterogeneity regarding sample collection methods, sequencing platforms, antibiotic exposure, and patient characteristics, standardized sampling time and processing protocols, analytical methods, and the uniform reporting of key clinical variables (such as antibiotic exposure, disease severity scores, and ICU type) are essential. This approach will significantly reduce methodological heterogeneity, improve result comparability, and promote the development of evidence-based clinical intervention strategies (Isali et al., 2024). Future studies must consistently and transparently report these key variables to allow for more comprehensive adjustment and robust analysis in subsequent meta-analyses.

## 5 Conclusion

Gut microbes are independently associated with mortality in critically ill patients, and early intervention for dysbiosis remains a promising therapeutic target in the ICU. Consistent with previous studies, we observed substantial compositional and diversity differences between ICU patients and healthy controls, with markedly reduced richness and diversity in the patient cohort.

With respect to sampling methodology, although some preliminary findings—such as the nonsignificant differences in *α*- diversity between fecal samples and rectal swabs—may suggest potential alternative sampling approaches, these results should be interpreted cautiously. The current evidence supporting direct equivalence between these two sampling methods is extremely limited and relies on only a small number of exploratory studies. Therefore, rectal swabs cannot yet be considered a universally optimal or reliable substitute for stool samples in gut microbiome or metabolome assessment among critically ill patients.

## Data Availability

The original contributions presented in the study are included in the article/Supplementary material, further inquiries can be directed to the corresponding author/s.

## Footnotes

1
http://www.math.hkbu.edu.hk/~tongt/papers/median2mean.html
