# Supplementary material for: Difference analysis of intestinal microbiota in patients in the intensive care unit using different sampling methods: a systematic review and meta-analysis
Source: Front Microbiol. 2026 Jan 23;16:1723862. doi: 10.3389/fmicb.2025.1723862 (PMC12880819; doi:10.3389/fmicb.2025.1723862)
Supplement: Supplementary file 1 [file Data_Sheet_1.pdf]

Supplement Table1. Systematic search detail.

Pubmed		
**Sequence**	**Search**	**Hits**
#1	("Gastrointestinal Microbiome"[Mesh]) OR (((((Gut microbiota) OR (Bacteria Community)) OR (Intestinal Microbiome)) OR (Intestinal Microbiota)) OR (Gut Flora))	221,521
#2	(((Fecal Processing Methods) OR (specimen processing method)) OR (rectal swab)) OR (stool samples)	246,926
#3	((("Gastrointestinal Microbiome"[Mesh]) OR (((((Gut microbiota) OR (Bacteria Community)) OR (Intestinal Microbiome)) OR (Intestinal Microbiota)) OR (Gut Flora))) and ((((Fecal Processing Methods) OR (specimen processing method)) OR (rectal swab)) OR (stool samples))) and (((comparison) OR (discrepancy)) OR (diversity))	4,372
#4	(((("Gastrointestinal Microbiome"[Mesh]) OR (((((Gut microbiota) OR (Bacteria Community)) OR (Intestinal Microbiome)) OR (Intestinal Microbiota)) OR (Gut Flora))) and ((((Fecal Processing Methods) OR (specimen processing method)) OR (rectal swab)) OR (stool samples))) and (((comparison) OR (discrepancy)) OR (diversity))) AND (ICU(Intensive Care Unit[Title/Abstract]))	46

web of science		
**Sequence**	**Search**	**Hits**
#1	TS=（Gut microbiota OR Gut Microbiome OR Bacteria Community OR Intestinal Microbiome OR Intestinal Microbiota OR Gut Flora）	214339
#2	TS=（Fecal Processing Methods OR specimen processing method OR rectal swab OR stool samples ）	64,737
#3	TS=(comparison OR discrepancy OR diversity ）	4,047,107
#4	TS=(ICU(Intensive Care Unit ））	56612
#5	1 and 2 and 3 and 4	63

Cochrane		
**Sequence**	**Search**	**Hits**
#1	MeSH descriptor: [Gastrointestinal Microbiome] explode all trees	1658
#2	(Gut microbiota OR Gut Microbiome OR Bacteria Community OR Intestinal Microbiome OR Intestinal Microbiota OR Gut Flora):ti,ab,kw	6580
#3	#1 OR #2	6936
#4	(Fecal Processing Methods OR specimen processing method OR rectal swab OR stool samples):ti,ab,kw	3804
#5	(comparison OR discrepancy OR diversity):ti,ab,kw	153435
#6	(Intensive Care Unit):ti,ab,kw	20034
#7	#3 and #4 and #5 and #6	8

Embase		
**Sequence**	**Search**	**Hits**
#1	'gut microbiota':ab,ti OR 'gut microbiome':ab,ti OR 'bacteria community':ab,ti OR 'intestinal microbiome':ab,ti OR 'intestinal microbiota':ab,ti OR 'gut flora':ab,ti	86604
#2	'fecal processing methods' OR (fecal AND ('processing'/exp OR processing) AND ('methods'/exp OR methods)) OR 'specimen processing method' OR (('specimen'/exp OR specimen) AND ('processing'/exp OR processing) AND ('method'/exp OR method)) OR 'rectal swab'/exp OR 'rectal swab' OR (rectal AND ('swab'/exp OR swab)) OR 'stool samples' OR (('stool'/exp OR stool) AND samples)	54856
#3	#1 and #2	8325
#4	'comparison'/exp OR comparison OR discrepancy OR 'diversity'/exp OR diversity	4134207
#5	'intensive care unit'/exp OR 'intensive care unit' OR (intensive AND ('care'/exp OR care) AND ('unit'/exp OR unit))	460842
#6	#3 and #4 and #5	111

CNKI（CNKI, China National Knowledge Internet.）		
**Sequence**	**Search**	**Hits**
#1	Gut microbiota OR Gut Microbiome OR Bacteria Community OR Intestinal Microbiome OR Intestinal Microbiota OR Gut Flora	43400
#2	#1 and Fecal Processing Methods OR specimen processing method OR rectal swab OR stool samples	11000
#3	#2 and comparison OR discrepancy OR diversity	224
#4	#3 and Intensive Care Unit	0

Supplementary Table 2 Crude Values of Microbial Diversity Indicators Comparing ICU Patients, Healthy Controls (HCs), and Specimen Types.

2.1 Crude Values of the Shannon Index Comparing ICU Patients and HCs.

Author	Year	m1	s1	n1	m2	s2	n2
Ekaterina Chernevskaya	2020	5.2	1.7	9	6.2	0.6	23
You-Dong Wan	2018	2.045	2	15	2.73	1.68	15
Xiaoya Zhang	2019	2.3084	0.98	16	3.3457	0.516	10
Antonio Mazzarelli	2021	2.92	1.6	6	3.3402	1.314	8
Xiangyu Long	2023	2.7214	2.2114	16	3.4249	1.7699	10

2.2 Crude Values of the Chao1 Index Comparing ICU Patients and HCs.

Author	Year	m1	s1	n1	m2	s2	n2
Antonio Mazzarelli	2021	184.38	140.71	6	251.09	134.72	8
Xiaoya Zhang	2019	158.1279	36.1299	16	231.1838	28.7758	10
Alexander Zaborin	2014	43	76.7706	31	84.184	15.5	5
Xiangyu Long 2023	2023	531.3447	542.404	16	867.6107	289.589	10

2.3 Crude Values of the Shannon Index Comparing Stool and Swab Samples in ICU Patients.

Author	Year	m1	s1	n1	m2	s2	n2
Katherine Fair	2019	1.6328	1.581	27	1.5613	2.1132	27
Sanmari é Schlebusch	2022	3.2	0.93	18	3.44	0.72	18
Saumya Bansal	2018	3.79	2.45	15	3.46	3.73	55

Note: n1, m1, s1: represent the sample size, mean and standard deviation in the experimental group; n2, m2, s2: represent the sample size, mean and standard deviation in the control group.

Supplement Figure

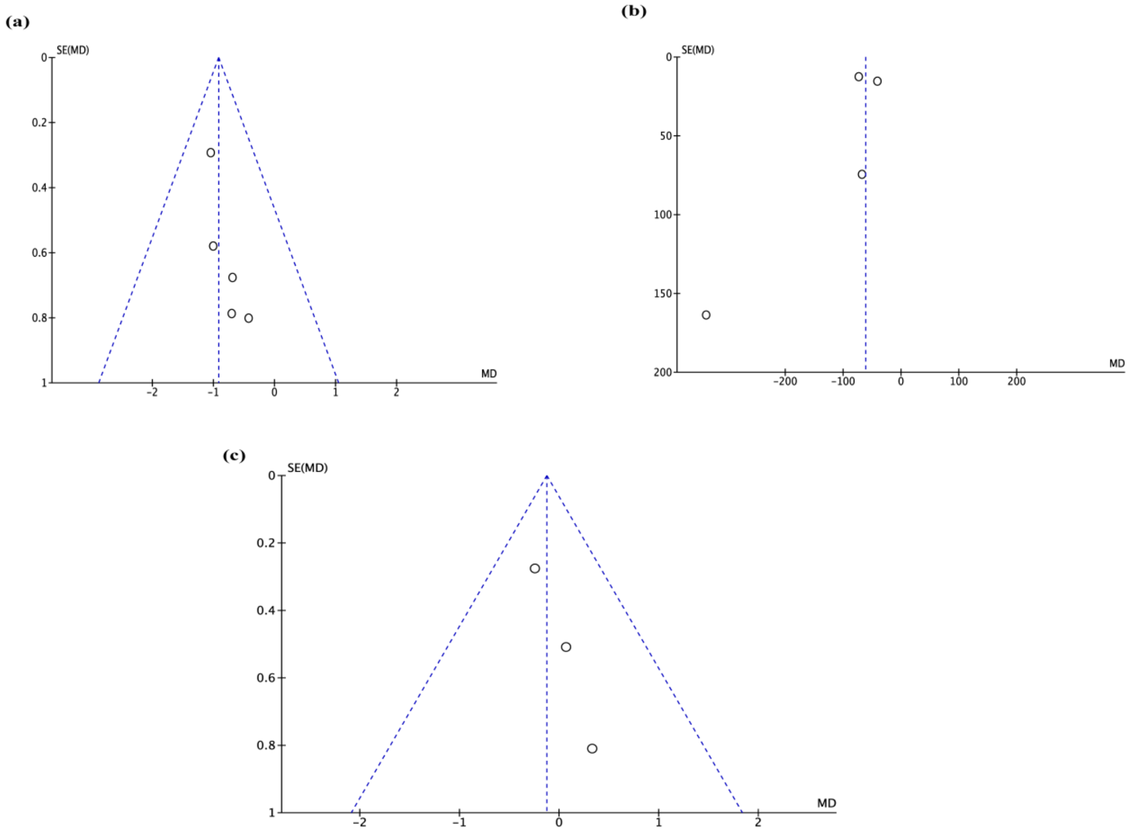

(a) Funnel plot of Shannon index. (b) Funnel plot of chao1 index. (c) Funnel plot of alpha diversity in Feces and swab samples from ICU patients.
